# Peritoneal catheter fixation versus nonfixation in open surgery: A meta-analysis

**DOI:** 10.1097/MD.0000000000051202

**Published:** 2026-10-16

**Authors:** Weigang Tang, Wei Jiang, Zhixia Wang, Chi Zhang, Tong Li, Xianping Li, Xiaoming Liu, Qing Luo, Macuo Wan, Wanxin Zhong, Zhuoma Caiji, Danzhi Hangxiu, Wei Zhao, Fancheng Kong, Wanping Zhou, Cuoji Yin, Xiang Dan, Zhanyin Yang, Jiali Qi, Guoyuan Lu, Wei Xu

**Affiliations:** a Department of Nephrology, The Wujin Clinical College of Xuzhou Medical University, Changzhou, Jiangsu, China; b Jiangsu Key Laboratory of New Drug Research and Clinical Pharmacy, Xuzhou Medical University, Xuzhou, Jiangsu, China; c Department of Nephrology, People’s Hospital of Hainan Tibetan Autonomous Prefecture, Hainan Tibetan Autonomous Prefecture, Qinghai, China; d Department of Nephrology, The First Affiliated Hospital of Soochow University, Suzhou, Jiangsu, China.

**Keywords:** Catheter fixation, complication, meta-analysis, peritoneal dialysis

## Abstract

**Background:**

The objective of this study was to compare the advantages and disadvantages of peritoneal dialysis (PD) catheter fixation versus nonfixation in open surgery for PD catheter placement.

**Methods:**

We searched the PubMed, Embase, Cochrane Library, China National Knowledge Infrastructure, and Wanfang databases from their inception to March 1, 2025. Eligible studies comparing PD catheter fixation with nonfixation in open surgery were included. The data were analyzed using Review Manager Version 5.3 and Stata Version 14.

**Results:**

Thirteen studies were included in the meta-analysis. A total of 648 patients were included in the PD catheter fixation group, and 627 patients were included in the PD catheter nonfixation group. Compared with PD catheter nonfixation, PD catheter fixation reduced the incidence of catheter displacement during open surgery (odds ratio [OR] 0.08, 95% confidence interval [CI] 0.04–0.16; *P* < .00001). However, there were no differences between the catheter fixation and catheter nonfixation groups concerning catheter malfunction without catheter displacement (OR 1.52, 95% CI 0.29–8.00, *P* = .62), omentum wrapping (OR 0.36, 95% CI 0.11–1.18, *P* = .09), infection (OR 0.95, 95% CI 0.51–1.78, *P* = .87), bleeding (OR 2.78, 95% CI 0.11–69.74, *P* = .53), infusion pain (OR 0.42, 95% CI 0.15–1.16, *P* = .10), or hernia (OR 0.66, 95% CI 0.11–4.07, *P* = .65). In addition, the operating time in the catheter fixation group was longer than that in the catheter nonfixation group (mean difference 4.18, 95% CI 1.42–6.94; *P* = .003).

**Conclusion:**

PD catheter fixation increased the operating time, but it reduced the incidence of catheter displacement and did not increase other complications in open surgery. PD catheter fixation is safe and effective in open surgery.

## 1. Introduction

Peritoneal dialysis (PD) is one of the best choices for renal replacement therapy in early end-stage kidney disease (ESKD).^[1,2]^ Because long-term treatment of PD patients requires a well-functioning PD catheter, appropriate PD catheterization technology is very important.^[3,4]^ PD catheter displacement is the most common complication that leads to catheter malfunction.^[5–7]^

Conventional open surgery has a high rate of PD catheter displacement.^[8–11]^ The laparoscopic PD catheter insertion technique is a good method that reduces the incidence of catheter displacement.^[12,13]^ However, the laparoscopic technique requires special equipment, general anesthesia, laparoscopic surgeons and has a high cost.^[14]^ A previous study indicated that modified catheter placement methods, such as a low incision site, a short intra-abdominal catheter segment, and an additional upwards straight subcutaneous tunnel, can reduce the incidence of catheter malfunction; however, patients still experienced catheter displacement.^[15]^ PD catheter placement, in which the intra-abdominal part of the catheter is fixed on the abdominal wall, has been recently reported to be a simple, economical, and effective method with a low incidence of catheter displacement. Therefore, this meta-analysis was conducted to evaluate the advantages and disadvantages of PD catheter fixation versus nonfixation in open surgery.

## 2. Materials and methods

### 2.1. Search strategy

Our meta-analysis was reported in accordance with the Preferred Reporting Items for Systematic Reviews and Meta-Analyses^[16]^ and the Assessing the Methodological Quality of Systematic Reviews guidelines.^[17]^ The Population, Interventions, Comparisons, and Outcomes strategy was developed to execute an accurate search strategy. The population included patients with ESKD who needed PD. The intervention studied was open surgery in which the intra-abdominal part of the PD catheter was fixed on the abdominal wall, bladder, or uterus. The comparison studied was open surgery in which the intra-abdominal part of the PD catheter was not fixed. The outcomes were catheter displacement, malfunction without catheter displacement, omentum wrapping, infection, bleeding, infusion pain, hernia, and operation time. Ethical approval was not necessary because our meta-analysis is a statistical analysis based on previous literature.

We searched the PubMed, Embase, Cochrane Library, China National Knowledge Infrastructure, and Wanfang databases from their inception to March 1, 2025. The keywords used were “peritoneal dialysis,” “catheter,” and “fixation.” In addition, the relevant references and cited publications were searched manually to identify additional studies meeting the inclusion criteria. There were no language restrictions.

### 2.2. Inclusion criteria

The inclusion criteria were as follows:

1. Randomized controlled trials (RCTs), cohort studies, or retrospective studies.
2. Studies on specific patients with ESKD who need dialysis.
3. Studies that compared PD catheter fixation with PD catheter nonfixation in open surgery. Open surgery can be performed by traditional placement techniques or low-site placement techniques.
4. Studies with outcomes that included catheter displacement, catheter malfunction without catheter displacement, omentum wrapping, infection (peritonitis, exit-site, or tunnel infection), bleeding, infusion pain, hernia, and operating time.

### 2.3. Exclusion criteria

The exclusion criteria were as follows:

1. Case series, comments, reviews.
2. No control group.
3. Lack of relevant outcome data.

### 2.4. Data extraction and quality assessment

Two independent researchers retrieved and selected all eligible reports. A 3rd investigator led the discussion if there were any disagreements. We extracted the following information: name of the 1st author, year of publication, location of the study, study design, follow-up period, sample size, sex, mean age, body mass index, history of abdominal disease, PD catheter placement method, and outcomes. Catheter displacement was defined as the tip of the catheter migrating out of the true pelvis. Catheter malfunction was defined as insufficient inflow and/or outflow of dialysate, including catheter tip migration and non-migration problems, which are mainly refractory obstructions. The Cochrane assessment tool was used to evaluate the quality of the RCTs,^[18]^ and the Newcastle–Ottawa scale was used to evaluate the quality of nonrandomized studies.^[19]^

### 2.5. Statistical analysis

We summarized treatment outcomes as odds ratios (ORs) for categorical variables and mean differences (MDs) for continuous variables with 95% confidence intervals (CIs). When the measurement units of continuous variables were different, the standardized mean difference (SMD) was selected to combine the effect size. We used the *I*^2^ statistic to assess heterogeneity among studies. We considered *I*^2^ > 50% and *P* < .10 to indicate significant heterogeneity. Meta-analyses with insignificant heterogeneity were performed using the fixed-effects model. The random-effects model was used for meta-analyses with significant heterogeneity. Publication bias was assessed using subgroup analysis, funnel plots, Egger test and sensitivity analysis. *P* < .05 was considered statistically significant. The data were analyzed using Review Manager Version 5.3 (The Nordic Cochrane Centre, Denmark) and Stata Version 14 (StataCorp LLC).

## 3. Results

### 3.1. Study selection and characteristics

The flow diagram of the systematic review is shown in Figure 1. A total of thirteen studies were included in the final analysis.^[20–32]^ Eight studies were cohort or retrospective studies, and 5 studies were RCTs. The traditional placement technique was used in 2 studies, and the low-site placement technique was used in 9 studies. The PD catheter was fixed to the bladder or uterus in 1 study, to the anterior abdominal wall in 11 studies, and to the lower abdominal wall in 1 study. Overall, 648 patients were included in the PD catheter fixation group, and 627 patients were included in the PD catheter nonfixation group. The follow-up period ranged from 1 to 35.3 months. The baseline characteristics of the thirteen studies are listed in Table 1. The risk of bias in the included RCTs was moderate. The nonrandomized studies with scores of ≥6 points were considered to be of high quality. The Cochrane assessments are listed in Table 2, and the Newcastle–Ottawa scale assessments are listed in Table 3.

**Table 1:** Characteristics of the included studies.

Study (year)	Country	Design	Follow-up period	Sample size	Mean age (yr)	Male (n, %)	BMI (kg/m^2^)	History of abdominal surgery, n (%)	PD catheter placement method	Catheter fixation position
Oka e al (2017)^[20]^	Japan	Rretrospective cohort study	12 mo	Fixation 17 Nonfixation 54	66 ± 5 63 ± 15	8 (47) 34 (63)	21 ± 3 21 ± 3	–	Traditional placement technique	Anterior abdominal wall
Io et al (2014)^[21]^	Japan	Cohort study	173.3 ± 73.5 d 273.2 ± 136.1 d	Fixation 25 Nonfixation 16	–	12 (75) 22 (88)	–	–	Traditional placement technique	Anterior abdominal wall
Wang et al (2014)^[22]^	China	Retrospective study	6 mo	Fixation 41 Nonfixation 52	53.5 52.3	19 (46) 25 (48)	–	0 (0) 1 (2)	Traditional placement technique	Anterior abdominal wall
Kume et al (2011)^[23]^	Japan	Retrospective study	23 mo 35.3 mo	Fixation 77 Nonfixation 75	–	–	21.8 22.5	9 (12) 13 (17)	Low-site placement technique	Bladder or uterus
Zhang et al (2018)^[24]^	China	RCT	6 mo	Fixation 4 Nonfixation 49	57.2 ± 16.6 55.9 ± 17.1	32 (59) 29 (59)	23.0 ± 1.8 22.5 ± 2.7	7 (13) 5 (10)	Low-site placement technique	Lower abdominal wall
Zhang and Wang (2009)^[25]^	China	Cohort study	1 mo	Fixation 62 Nonfixation 46	52.2 ± 0.5 50.8 ± 0.5	56 (90) 44 (96)	–	–	Traditional placement technique	Anterior abdominal wall
Chen and Dong (2008)^[26]^	China	RCT	12 mo	Fixation 35 Nonfixation 28	40.5 ± 9.6 38.8 ± 9.6	18 (51) 15 (54)	–	–	Traditional placement technique	Anterior abdominal wall
Zhu et al (2019)^[27]^	China	Cohort study	6 mo	Fixation 155 Nonfixation 123	51.5 55.3	85 (55) 65 (53)	–	–	Traditional placement technique	Anterior abdominal wall
Lu et al (2013)^[28]^	China	RCT	21 mo	Fixation 24 Nonfixation 24	53	30 (63)	–	–	Traditional placement technique	Anterior abdominal wall
Xu (2015)^[29]^	China	Cohort study	–	Fixation 32 Nonfixation 32	43.2 ± 2.4 44.2 ± 2.2	19 (59) 18 (56)	–	–	Traditional placement technique	Anterior abdominal wall
Zhang et al (2021)^[30]^	China	RCT	6 mo	Fixation 60 Nonfixation 60	50.8 ± 13.9 50.2 ± 12.4	35 (58) 38 (63)	22.5 ± 3.4 23.3 ± 3.3	–	Traditional placement technique	Anterior abdominal wall
Hu et al (2017)^[31]^	China	Cohort study	12–18 mo	Fixation 48 Nonfixation 48	44.8 ± 4.6 43.6 ± 4.8	22 (46) 20 (42)	–	–	Traditional placement technique	Anterior abdominal wall
Wang et al (2008)^[32]^	China	RCT	1 mo	Fixation 18 Nonfixation 20	54.3	17 (47)	–	–	Traditional placement technique	Anterior abdominal wall

BMI = body mass index, PD = peritoneal dialysis, RCT = randomized controlled trial.

**Table 2:** Quality assessment of randomized control trial.

Study	Random sequence generation	Allocation concealment	Blinding of participants and personnel	Incomplete outcome data	Selective reporting	Other bias
Zhang et al (2018)^[24]^	+	?	?	+	+	?
Chen and Dong (2008)^[26]^	?	?	?	+	+	?
Lu et al (2013)^[28]^	?	?	?	+	+	?
Zhang et al (2021)^[30]^	?	?	?	+	+	?
Wang et al (2008)^[32]^	+	?	?	+	+	?

The randomized control trial was evaluated using the Cochrane assessment tool.

+ = low risk of bias, ? = unclear risk of bias, - = high risk of bias.

**Table 3:** Quality assessment of nonrandomized studies.

Studies	Selection	Comparability	Outcome	Score
Oka et al (2017)^[20]^	**★★★**	**★**	**★★★**	7
Io et al (2014)^[21]^	**★★★**	**★**	**★★**	6
Wang et al (2014)^[22]^	**★★★**	**★**	**★★**	6
Kume et al (2011)^[23]^	**★★★**	**★**	**★★★**	7
Zhang and Wang (2009)^[25]^	**★★★★**	**★**	**★★**	7
Zhu et al (2019)^[27]^	**★★★**	**★**	**★★★**	7
Xu (2015)^[29]^	**★★★**	**★**	**★★**	6
Hu et al (2017)^[31]^	**★★★**	**★**	**★★**	6

The cohort studies were evaluated using the Newcastle–Ottawa scale,which is comprised of the study of selection (representativeness of the exposed group, representativeness of the non-exposed group, ascertainment of exposure, and demonstration that outcome of interest was not present at start of study), group comparability (controls for the most important factor and controls for any additional factor), and outcome measures (assessment of outcome, was follow-up long enough for outcomes to occur, and adequacy of follow-up of cohorts), for a total of 9 points.

**★** = 1 point.

**Figure 1.**
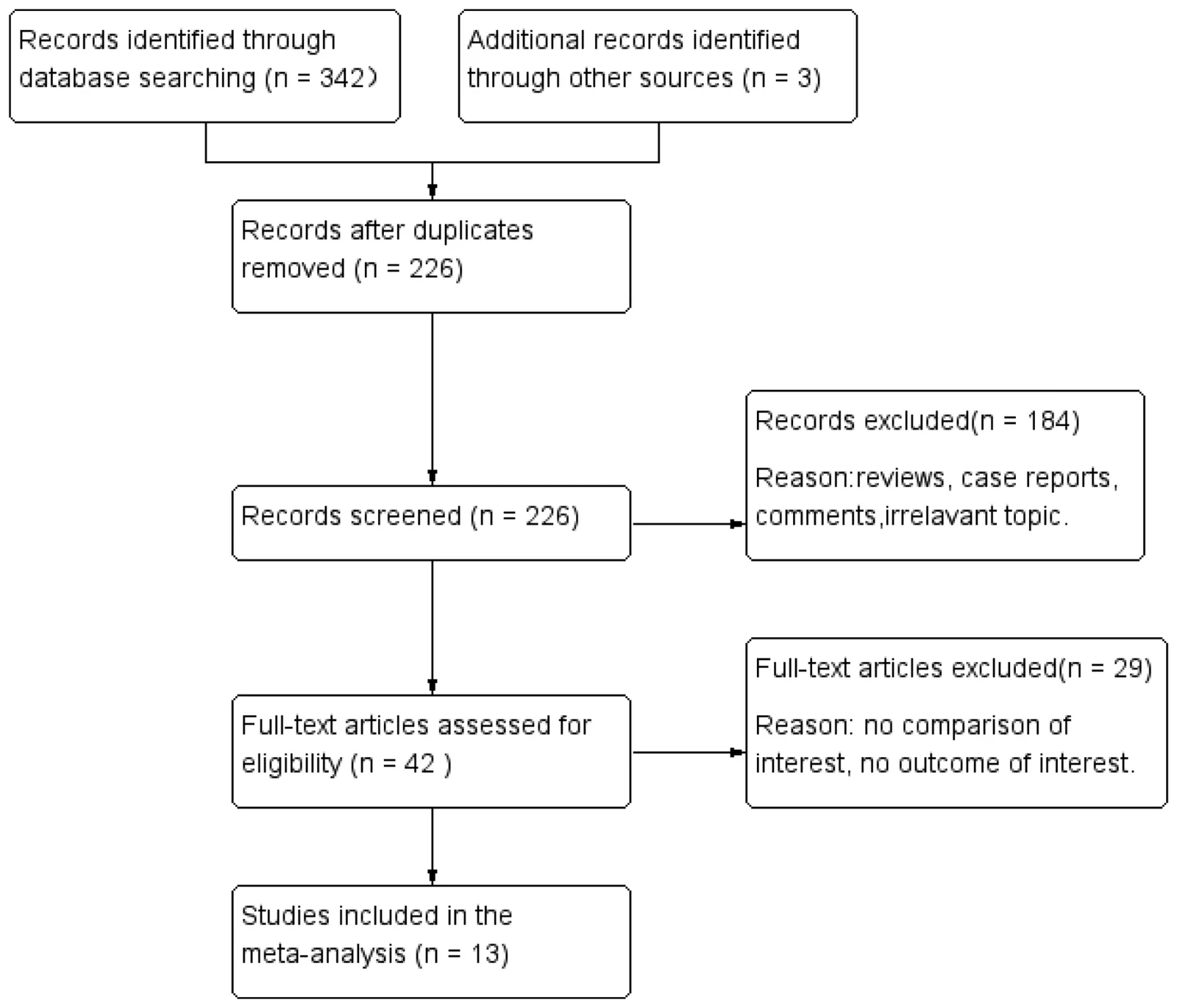
Flow diagram of the literature search.

### 3.2. Meta-analysis results

#### 3.2.1. Catheter displacement

Data about catheter displacement were reported in all studies. Owing to the insignificant heterogeneity of the included studies, the fixed-effects model was adopted. A subgroup analysis was performed according to the method of open surgery. In the traditional placement technique subgroup, the incidence of catheter displacement in the catheter fixation group was lower than that in the catheter nonfixation group (OR 0.08, 95% CI 0.04–0.17; *P* < .00001). In the low-site placement technique subgroup, the incidence of catheter displacement in the catheter fixation group was lower than that in the catheter nonfixation group (OR 0.08, 95% CI 0.01–0.63; *P* = .02). Overall, the incidence of catheter displacement in the catheter fixation group was lower than that in the catheter nonfixation group (OR 0.08, 95% CI 0.04–0.16; *P* < .00001) (Fig. 2).

**Figure 2.**
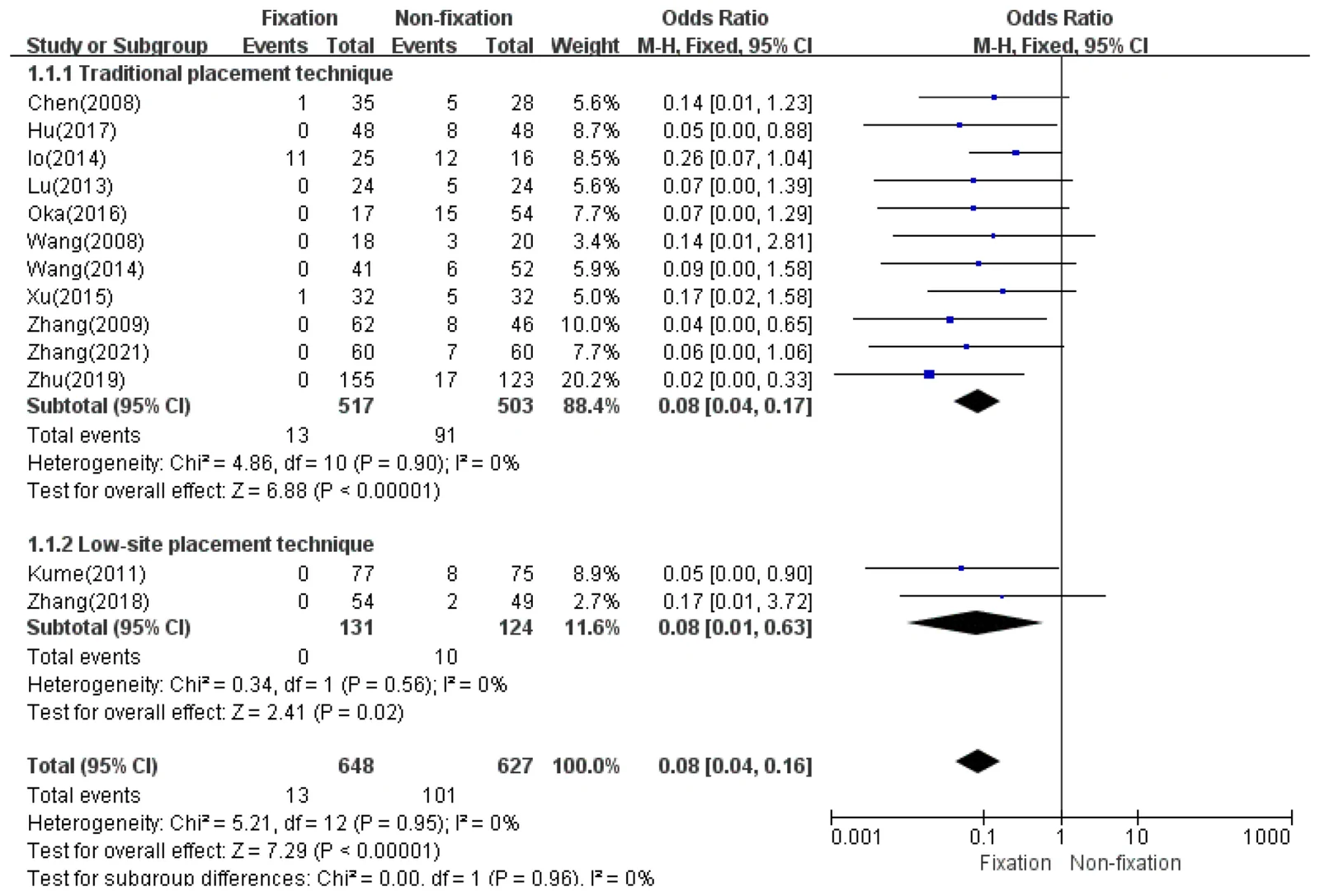
Forest plots comparing the incidence of catheter displacement between catheter fixation and nonfixation in open surgery. CI = confidence interval.

#### 3.2.2. Catheter malfunction without catheter displacement

Data concerning catheter malfunction without catheter displacement were reported in 3 studies. Owing to the insignificant heterogeneity of the included studies, the fixed-effects model was adopted. A subgroup analysis was performed according to the method of open surgery. In the traditional placement technique subgroup, there was no significant difference between the catheter fixation group and the catheter nonfixation group concerning the incidence of catheter malfunction without catheter displacement (OR 0.41, 95% CI 0.02–10.42; *P* = .59). In the low-site placement technique subgroup, there was no significant difference in the incidence of catheter malfunction without catheter displacement between the catheter fixation group and the catheter nonfixation group (OR 3.00, 95% CI 0.31–29.51; *P* = .35). When the results of all the subgroups were summarized, there was no significant difference in the incidence of catheter malfunction without catheter displacement between the catheter fixation group and the catheter nonfixation group (OR 1.52, 95% CI 0.29–8.00; *P* = .62). (Fig. 3).

**Figure 3.**
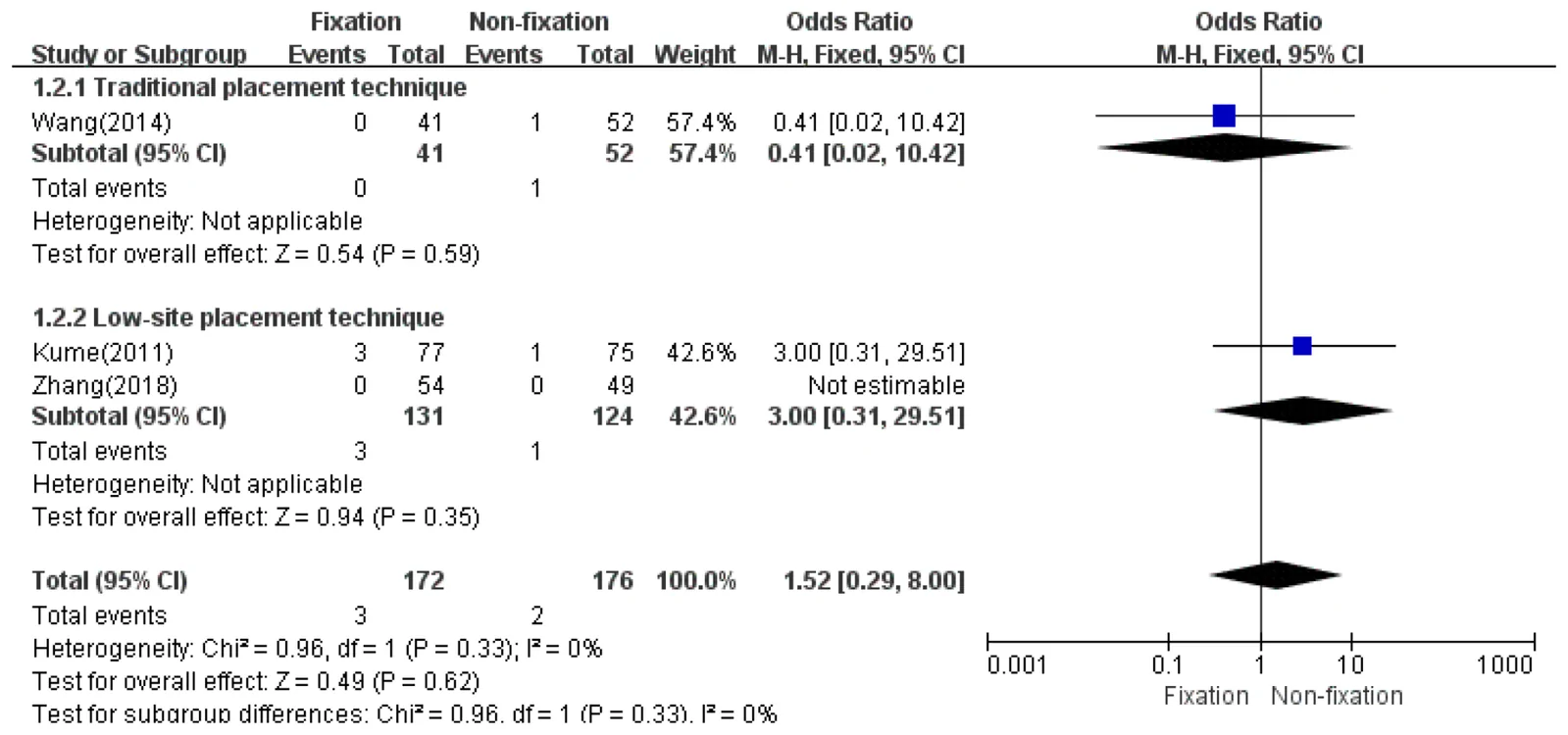
Forest plots comparing the incidence of catheter malfunction without catheter displacement between catheter fixation and nonfixation in open surgery. CI = confidence interval.

#### 3.2.3. Omentum wrap

Data concerning the presence of omentum wrapping were reported in 3 studies. Owing to the insignificant heterogeneity of the included studies, the fixed-effects model was adopted. In traditional open surgery, there was no significant difference in the incidence of omentum wrapping between the catheter fixation group and the catheter nonfixation group (OR 0.36, 95% CI 0.11–1.18; *P* = .09) (Fig. 4).

**Figure 4.**
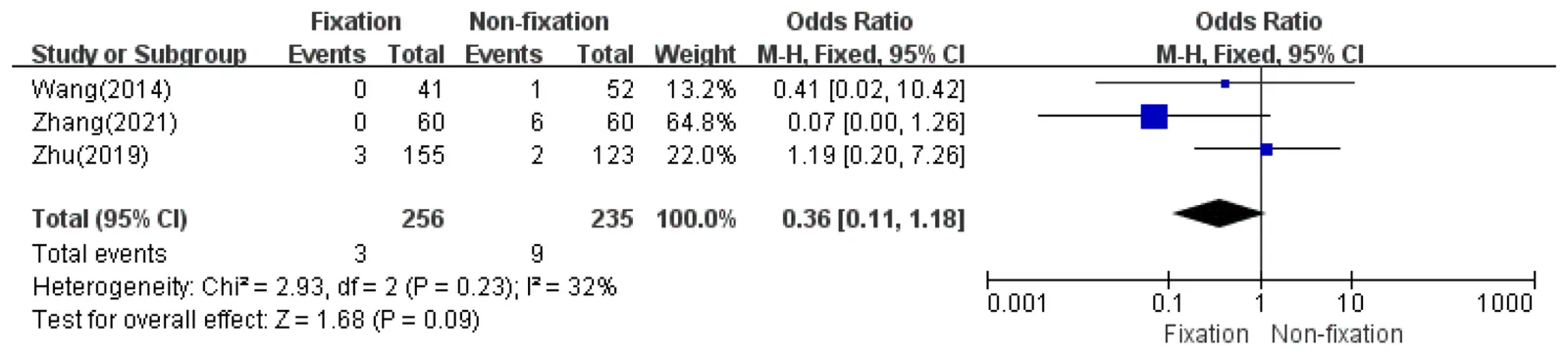
Forest plots comparing the incidence of omentum wrap between catheter fixation and nonfixation in traditional open surgery. CI = confidence interval.

#### 3.2.4. Infection

Data about infection were reported in 6 studies. Owing to the insignificant heterogeneity of the included studies, the fixed-effects model was adopted. A subgroup analysis was performed according to the method of open surgery. In the traditional placement technique subgroup, there was no significant difference in the incidence of infection between the catheter fixation group and the catheter nonfixation group (OR 0.90, 95% CI 0.45–1.79; *P* = .77). In the low-site placement technique subgroup, there was no significant difference in the incidence of infection between the catheter fixation group and the catheter nonfixation group (OR 1.23, 95% CI 0.26–5.78; *P* = .80). When the results of all the subgroups were summarized, there was no significant difference in the incidence of infection between the catheter fixation group and the catheter nonfixation group (OR 0.95, 95% CI 0.51–1.78; *P* = .87). (Fig. 5).

**Figure 5.**
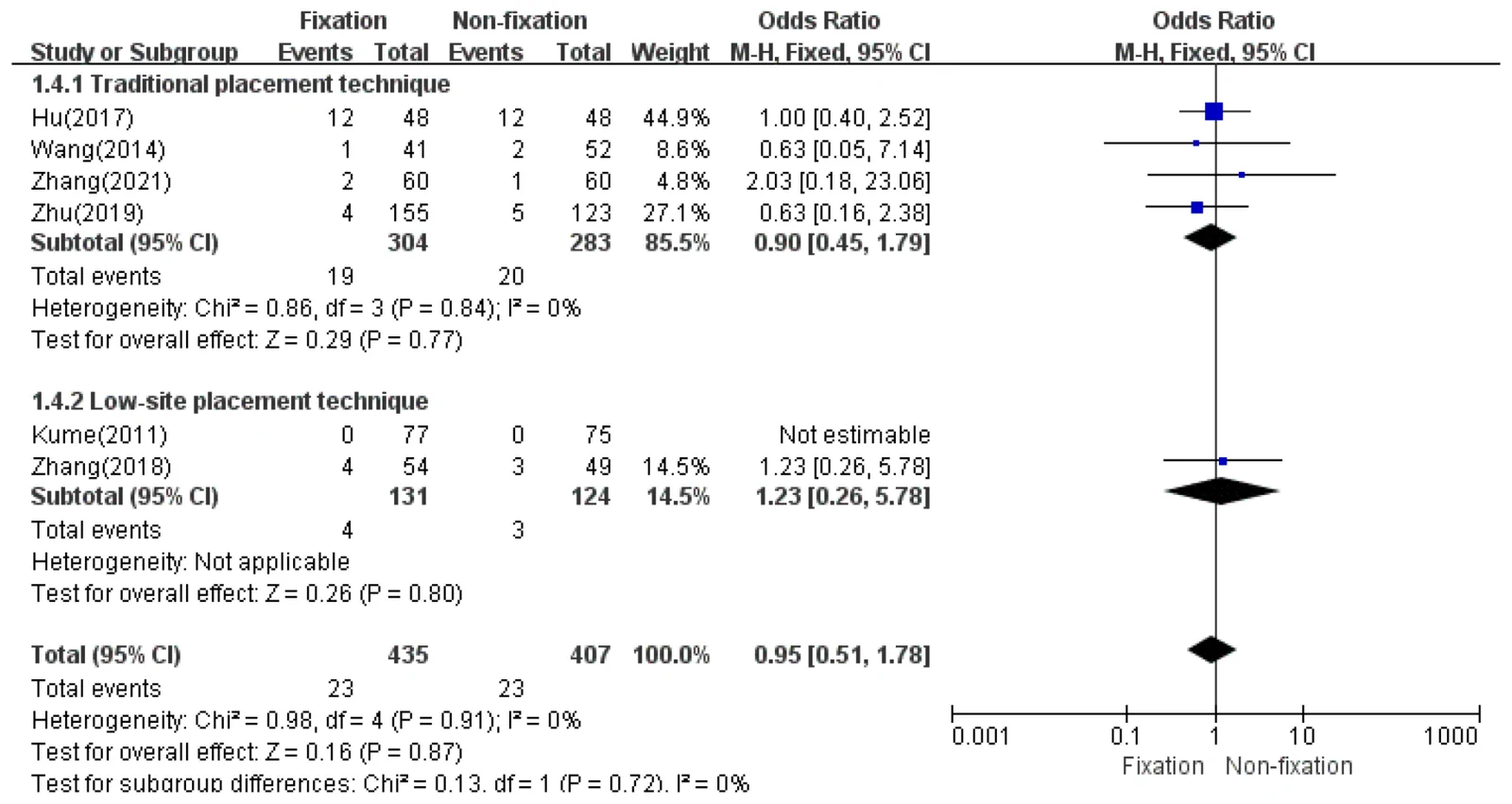
Forest plots comparing the incidence of infection between catheter fixation and nonfixation in open surgery. CI = confidence interval.

#### 3.2.5. Bleeding

Data about bleeding were reported in 2 studies. A subgroup analysis was performed according to the method of open surgery. In the traditional placement technique subgroup, there was no bleeding during catheter fixation or in the catheter nonfixation group. In the low-site placement technique subgroup, there was no significant difference in the incidence of bleeding between the catheter fixation group and the catheter nonfixation group (OR 2.78, 95% CI 0.11–69.74; *P* = .53). When the results of all the subgroups were summarized, there was no significant difference in the incidence of catheter malfunction without catheter displacement between the catheter fixation group and the catheter nonfixation group (OR 2.78, 95% CI 0.11–69.74; *P* = .53). (Fig. 6).

**Figure 6.**
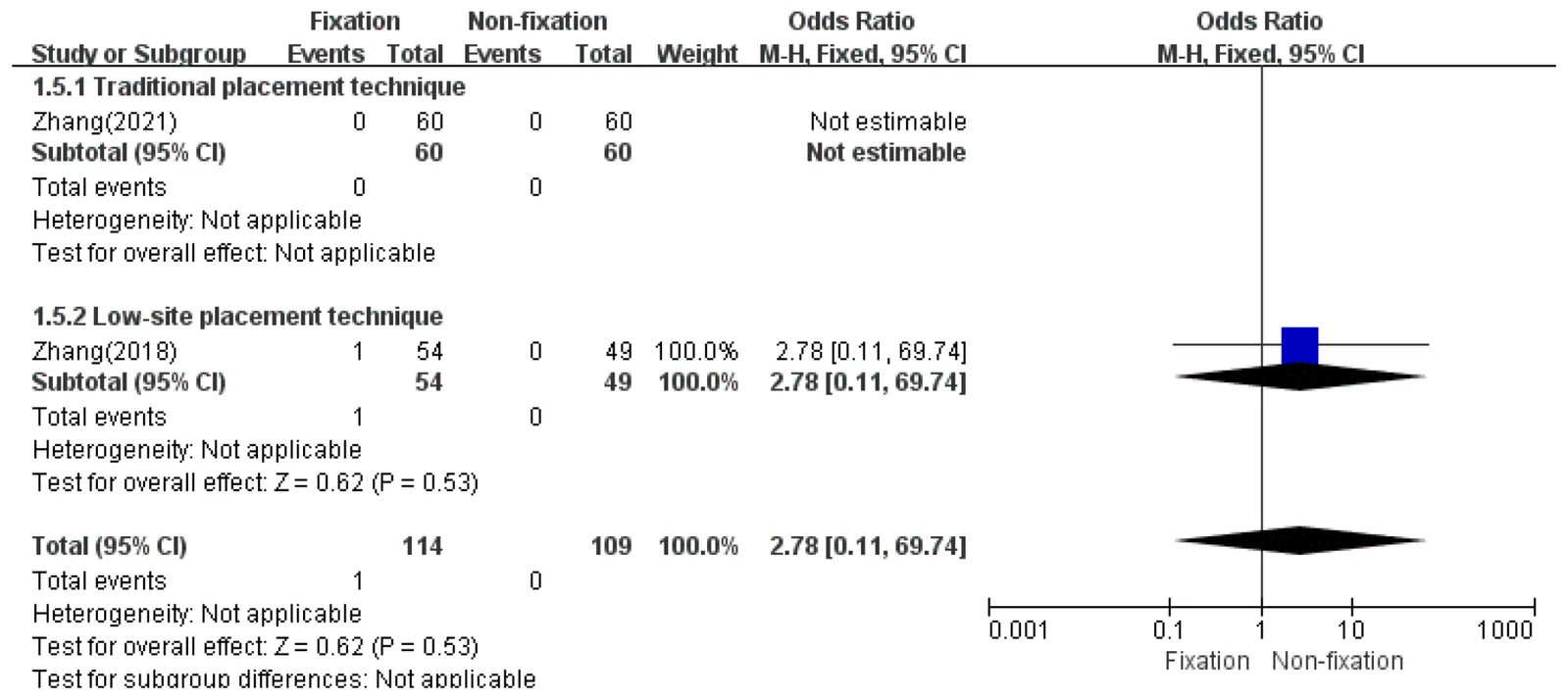
Forest plots comparing the incidence of bleeding between catheter fixation and nonfixation in open surgery. CI = confidence interval.

#### 3.2.6. Infusion pain

Data concerning infusion pain were reported in 3 studies. Owing to the insignificant heterogeneity of the included studies, the fixed-effects model was adopted. In traditional open surgery, there was no significant difference in the incidence of infusion pain between the catheter fixation group and the catheter nonfixation group (OR 0.42, 95% CI 0.15–1.16; *P* = .10) (Fig. 7).

**Figure 7.**
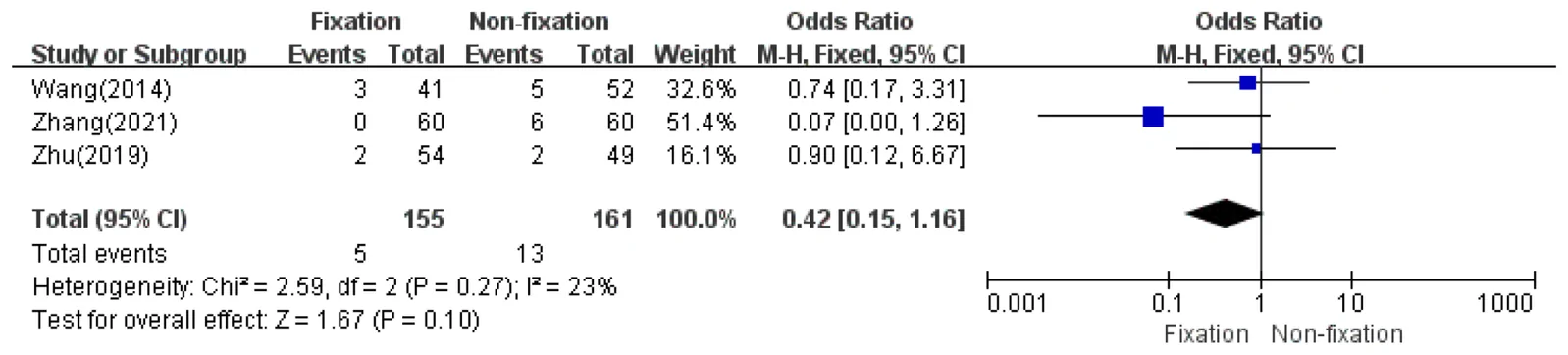
Forest plots comparing the incidence of infusion pain between catheter fixation and nonfixation in traditional open surgery. CI = confidence interval.

#### 3.2.7. Hernia

Data concerning hernias were reported in 2 studies. A subgroup analysis was performed according to the method of open surgery. In the traditional placement technique subgroup, there was no significant difference in the incidence of hernia between the catheter fixation group and the catheter nonfixation group (OR 0.66, 95% CI 0.11–4.07; *P* = .65). In the low-site placement technique subgroup, there was no hernia in the catheter fixation or in the catheter nonfixation group. When the results of all the subgroups were summarized, there was no significant difference in the incidence of hernia between the catheter fixation group and the catheter nonfixation group (OR 0.66, 95% CI 0.11–4.07; *P* = .65) (Fig. 8).

**Figure 8.**
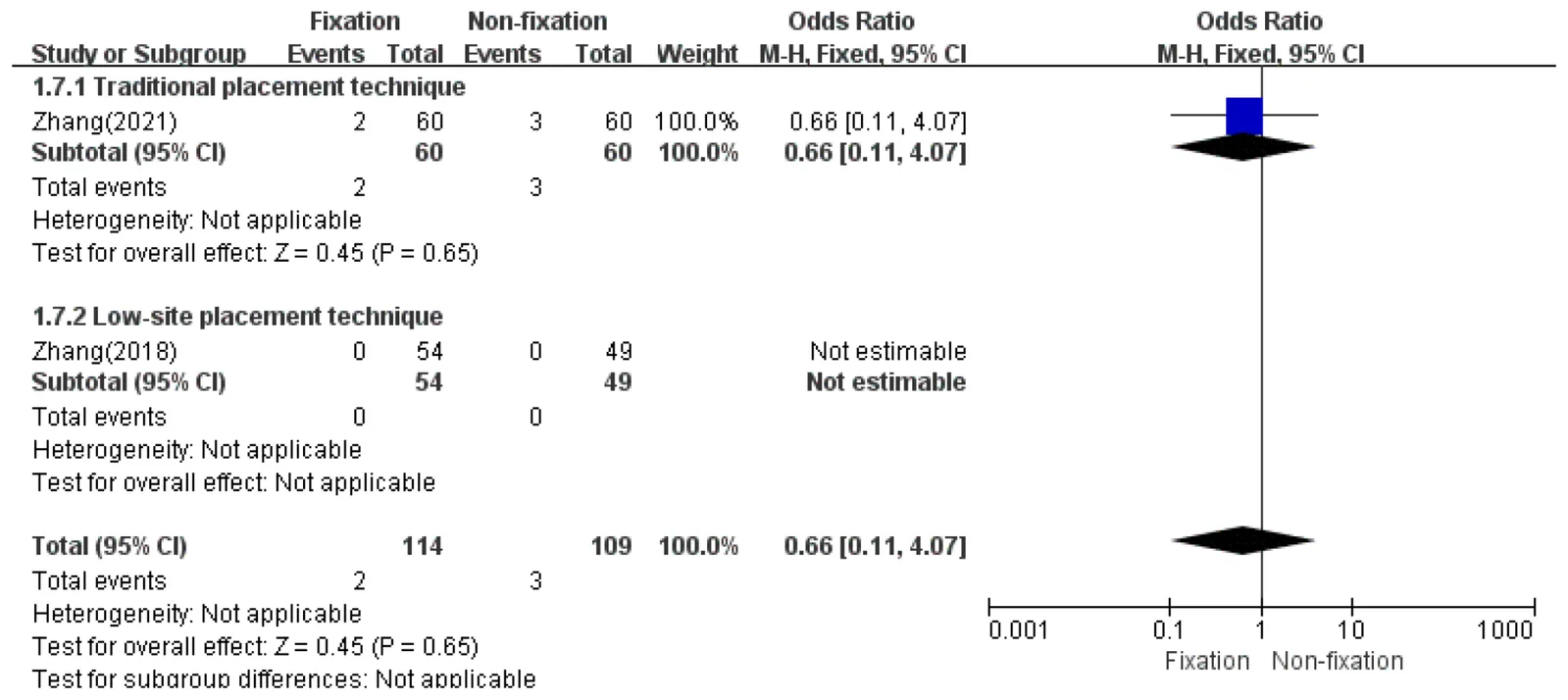
Forest plots comparing the incidence of hernia between catheter fixation and nonfixation in open surgery. CI = confidence interval.

#### 3.2.8. Operating time

The data concerning operating time were reported in 4 articles. Owing to the significant heterogeneity of the included studies, a random-effects model was adopted. A subgroup analysis was performed according to the method of open surgery. In the traditional placement technique subgroup, the operating time in the catheter fixation group was longer than that in the catheter nonfixation group (MD 4.65, 95% CI 0.23–9.08; *P* = .04). In the low-site placement technique subgroup, the operating time in the catheter fixation group was also longer than that in the catheter nonfixation group (MD 3.80, 95% CI 0.71–6.89; *P* = .02). When the results of all the subgroups were summarized, the operating time in the catheter fixation group was also longer than that in the catheter nonfixation group (MD 4.18, 95% CI 1.42–6.94, *P* = .003) (Fig. 9).

**Figure 9.**
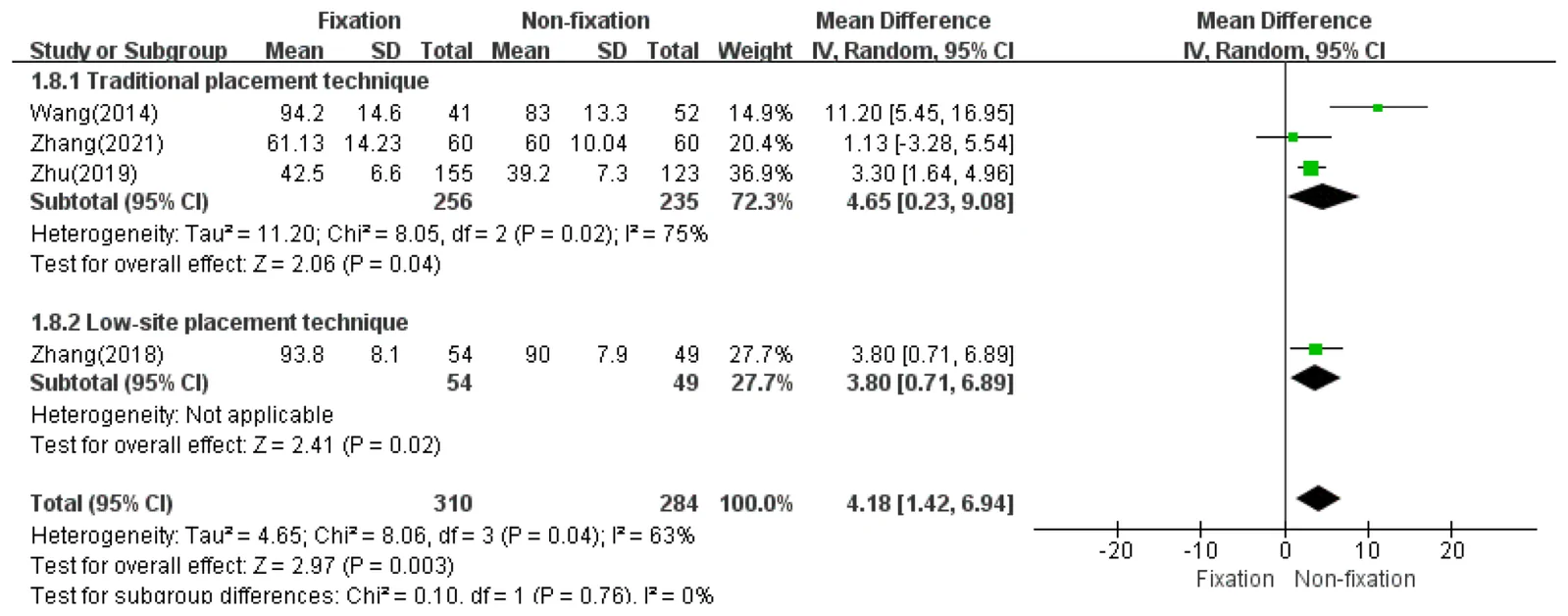
Forest plots comparing operating time between catheter fixation and nonfixation in open surgery. CI = confidence interval, SD = standard deviation.

### 3.3. Funnel plot and sensitivity analyses

Funnel plots were used to depict publication bias concerning the meta-analysis results of catheter displacement. The funnel plot showed possible asymmetries, which might indicate that there was potential publication bias in the meta-analysis results of catheter displacement (Fig. 10). Egger test also indicated that there was potential publication bias in the meta-analysis results of catheter displacement (*P* < .05). Sensitivity analyses were used to judge the dependability of the meta-analysis results for other outcomes. We excluded one of the studies at a time, and the results of catheter malfunction without catheter displacement, omentum wrapping, infection, bleeding, infusion pain, and hernia still showed no difference. When we excluded the studies of Zhang (2021) or Zhu (2019), there was no significant difference in operating time between the catheter fixation group and the catheter nonfixation group.

**Figure 10.**
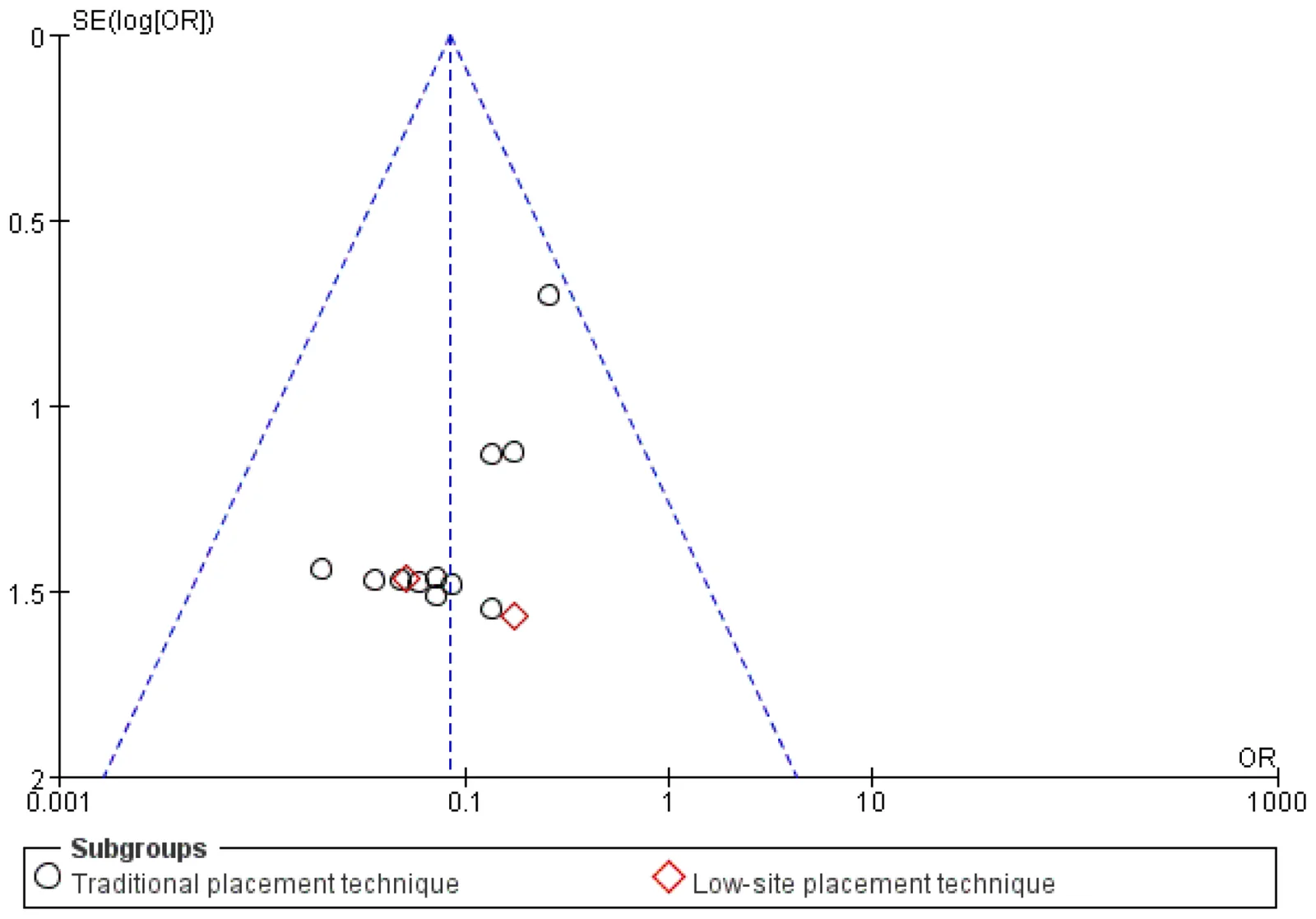
Funnel plots concerning the meta-analysis result of catheter displacement. OR = odds ratio, SE = standard error.

## 4. Discussion

Long-term safe and effective access is the key to successful PD.^[33]^ The current incidence of catheter-related complications in traditional open surgery is high, especially the incidence of catheter displacement, which is 10% to 22%.^[9–11]^ Our meta-analysis revealed that the technique of PD catheter fixation reduced the incidence of catheter displacement compared with PD catheter nonfixation in open surgery. However, there were no differences between the catheter fixation and nonfixation groups concerning catheter malfunction without catheter displacement, omentum wrapping, infection, bleeding, infusion pain, or hernia. In addition, the operating time in the catheter fixation group was longer than that in the catheter nonfixation group.

Catheter displacement is defined as the tip of the catheter migrating out of the true pelvis, which can cause outflow obstruction and is one of the main catheter-related complications in traditional open surgery.^[34]^ The results of our meta-analysis revealed that PD catheter fixation reduced the incidence of catheter displacement in open surgery. For the traditional placement technique, the peritoneal opening is at a high position, and the catheter is fixed in the anterior peritoneum. For the low-position placement technique, the peritoneal opening is placed at a low position, and the catheter is fixed in the lower abdominal wall, bladder, or uterus. Regardless of whether the fixed position of the catheter was high or low, the incidence of catheter displacement decreased. In addition, the low-site placement technique can reduce the incidence of catheter displacement.^[2]^ However, our results showed that low-site placement with PD catheter fixation can further reduce the incidence of catheter displacement compared with low-site placement without PD catheter fixation. In all the included studies, the intra-abdominal part of the PD catheter was fixed on the abdominal wall, bladder, or uterus, which reduced the length of the catheter-free segment in the abdominal cavity.^[35]^ Thus, the possibility of catheter displacement was greatly reduced. Even with a small movement of the catheter, it is difficult for the catheter to be removed from the true pelvis.^[31]^ It should be highlighted that the purpose of intra-abdominal catheter fixation is to prevent upward migration, rather than to create rigid and tight anchorage onto the abdominal wall. According to the Japanese peritoneal dialysis guidelines, in the PD catheter insertion procedure, the catheter should be smoothly slid along the abdominal wall until it reaches the Douglas pouch.^[6,36]^ Correct placement within the Douglas pouch is the foundation for ensuring the smooth flow of dialysis fluid in and out. Catheter fixation serves as an auxiliary protective measure to prevent the catheter tip from protruding out of the true pelvis, and maintaining the optimal position within the pelvis remains the main surgical objective.

Laparoscopic catheter placement is an established alternative that also effectively reduces catheter displacement. Nevertheless, laparoscopic intervention requires dedicated endoscopic equipment, general anesthesia, and surgeons with advanced laparoscopic training, which may not be accessible in many resource-limited regions.^[14]^ By comparison, open-surgical catheter fixation is implemented under conventional open conditions without high-end instruments. It represents a low-cost, practical option for centers where laparoscopic resources are scarce.

Our meta-analysis demonstrated that the technique of PD catheter fixation significantly reduced the incidence of catheter displacement at the expense of longer operative duration. This trade-off deserves careful clinical interpretation. Although several extra minutes of operative time are added during PD catheter fixation, catheter displacement is a troublesome complication that frequently leads to dialysate flow failure, repeated radiological reposition attempts, hospital readmission, and even permanent catheter loss for ESKD patients. Avoiding these adverse events may offset the disadvantage of moderately prolonged surgery from the perspective of patient-centered outcomes.

The extra procedure of PD catheter fixation by suture still requires careful operation by the surgeon, especially to avoid bleeding, organ damage, and infection. In our meta-analysis, the extra procedure of catheter fixation increased the surgical operating time, but it did not increase the incidence of other complications, such as catheter malfunction without catheter displacement, omentum wrapping, infection, bleeding, infusion pain, or hernia. Although the data concerning organ injury were reported in 2 included studies, we did not perform a meta-analysis because no organ injury occurred during the operation of PD catheter fixation or nonfixation.^[24,30]^ These findings indicate that PD catheter fixation is a safe and effective technique in open surgery. Furthermore, it is worth noting that several improved types of PD catheters have been developed. For instance, 1 of them has the feature of a thicker section near the peritoneal cuff, which aims to enhance the fixation performance of the catheter and potentially reduce the risks of catheter displacement and leakage around the catheter. In the future, we can conduct further research in this area.

Our meta-analysis had several limitations. Firstly, only 5 RCTs were included among 13 eligible studies. The small proportion of RCTs limits the strength and certainty of the evidence. Secondly, funnel-plot asymmetry and Egger test suggested potential publication bias for the outcome of catheter displacement. Publication bias may overestimate the protective effect of catheter fixation against catheter displacement, as small-sized studies with neutral or negative results might remain unpublished. Thirdly, there are still differences in terms of the fixed position, the suture location, and the length of the abdominal cavity catheter among included studies. Therefore, future RCTs are urgently required to develop and implement standardized fixation protocols. Uniform surgical definitions and procedural descriptions will help produce more robust, reproducible evidence to guide routine clinical practice.In addition,the risk of omentum wrapping may be influenced by the characteristics of the catheter-related hardware, including the type of catheter and the size of the side holes. These details regarding the catheter vary among the studies included in our meta-analysis, and they may become unmeasured confounding factors when interpreting the results of omentum wrapping we have summarized.

## 5. Conclusions

PD catheter fixation is safe and effective in open surgery. PD catheter fixation increased the operating time but reduced the incidence of catheter displacement in patients who underwent open surgery. Moreover, PD catheter fixation did not increase the incidence of catheter malfunction without catheter displacement, omentum wrapping, infection, bleeding, infusion pain, or hernia.

## Author contributions

**Conceptualization:** Weigang Tang, Wei Xu.

**Data curation:** Wei Jiang, Zhixia Wang.

**Investigation:** Chi Zhang, Tong Li, Xianping Li.

**Methodology:** Weigang Tang, Wei Xu.

**Supervision:** Weigang Tang, Guoyuan Lu, Wei Xu.

**Visualization:** Wei Zhao, Fancheng Kong, Xiang Dan, Zhanyin Yang.

**Writing – original draft:** Xiaoming Liu, Qing Luo, Macuo Wan, Wanxin Zhong, Zhuoma Caiji, Danzhi Hangxiu, Wanping Zhou, Cuoji Yin, Jiali Qi.
